# Magnetized chitosan hydrogel and silk fibroin, reinforced with PVA: a novel nanobiocomposite for biomedical and hyperthermia applications

**DOI:** 10.1039/d3ra00612c

**Published:** 2023-03-14

**Authors:** Reza Eivazzadeh-Keihan, Zeinab Pajoum, Hooman Aghamirza Moghim Aliabadi, Adibeh Mohammadi, Amir Kashtiaray, Milad Salimi Bani, Banafshe Pishva, Ali Maleki, Majid M. Heravi, Mohammad Mahdavi, Elaheh Ziaei Ziabari

**Affiliations:** a Catalysts and Organic Synthesis Research Laboratory, Department of Chemistry, Iran University of Science and Technology Tehran 16846-13114 Iran maleki@iust.ac.ir +98-21-73021584 +98-21-73228313; b Department of Chemistry, School of Physics and Chemistry, Alzahra University PO Box 1993891176, Vanak Tehran Iran mmheravi@alzahra.ac.ir; c Advanced Chemical Studies Lab, Department of Chemistry, K. N. Toosi University of Technology Tehran Iran; d Department of Biomedical Engineering, Faculty of Engineering, University of Isfahan Isfahan Iran; e Endocrinology and Metabolism Research Center, Endocrinology and Metabolism Clinical Sciences Institute, Tehran University of Medical Sciences Tehran Iran momahdavi@sina.tums.ac.ir; f Department of Orthopedic Surgery, Rothman Institute, Thomas Jefferson University 125 South 9th Street, Suite 1000 Philadelphia PA 19107 USA

## Abstract

Herein, a multifunctional nanobiocomposite was designed for biological application, amongst which hyperthermia cancer therapy application was specifically investigated. This nanobiocomposite was fabricated based on chitosan hydrogel (CS), silk fibroin (SF), water-soluble polymer polyvinyl alcohol (PVA) and iron oxide magnetic nanoparticles (Fe_3_O_4_ MNPs). CS and SF as natural compounds were used to improve the biocompatibility, biodegradability, adhesion and cell growth properties of the nanobiocomposite that can prepare this nanocomposite for the other biological applications such as wound healing and tissue engineering. Since the mechanical properties are very important in biological applications, PVA polymer was used to increase the mechanical properties of the prepared nanobiocomposite. All components of this nanobiocomposite have good dispersion in water due to the presence of hydrophilic groups such as NH_2_, OH, and COOH, which is one of the effective factors in increasing the efficiency of hyperthermia cancer therapy. The structural analyzes of the hybrid nanobiocomposite were determined by FT-IR, XRD, EDX, FE-SEM, TGA and VSM. Biological studies such as MTT and hemolysis testing proved that it is hemocompatible and non-toxic for healthy cells. Furthermore, it can cause the death of cancer cells to some extent (20.23%). The ability of the nanobiocomposites in hyperthermia cancer therapy was evaluated. Also, the results showed that it can be introduced as an excellent candidate for hyperthermia cancer therapy.

## Abstract 2

Herein, a multifunctional nanobiocomposite was designed for biological application, amongst which hyperthermia cancer therapy application was specifically investigated.

## Graphical abstract

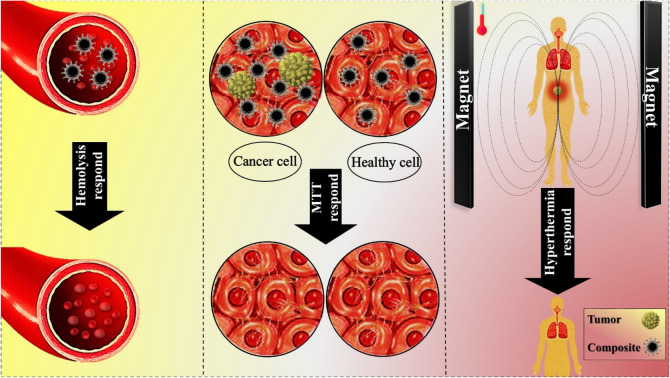

## 1. Introduction

Hydrogels are three-dimensional polymer networks that absorb water and other biological fluids due to the presence of hydrophilic functional groups such as amines, amides, hydroxyl, and sulfates in their structure.^1^ The first use of hydrogels dates back to 1949 when poly(vinyl alcohol) cross-linked with formaldehyde was used for biomedical implants.^2^ However, the starting point and spread of hydrogels can be attributed to the synthesis of poly(2-hydroxyethyl methacrylate) gels used for contact lenses.^3^ Hydrogels are one of the most widely used and ideal materials in medical science due to their hydrophilic nature, versatile fabrication platforms into materials, elasticity, high biocompatibility, as well as their ability to carry drugs, proteins, growth factors, and small molecules.^4^ They can be divided into three generations: the first generation is hydrogels with high strength and flexibility, which are mainly acquired by the mechanism of chain addition reaction and include vinyl monomers and a single free-starter species. The most common polymers in this group are polyacrylamide, poly(hydroxy-alkyl methacrylate) (pHEMA), polyvinyl alcohol (PVA), polyethylene glycol (PEG), and cellulose, which have been mainly used in the formation of gels for agriculture, contact lenses, drug delivery, and tissue engineering.^5^ The second generation is stimulus-responsive hydrogels that can respond to specific stimuli such as temperature, pH, or biological molecules.^6^ The use of these polymers led to an important event in targeted drug delivery. These hydrogels usually have acidic or basic segments that can be hydrolyzed at low or high pH. The third generation also includes stereo-complex materials such as poly(lactic acid)–poly(ethylene glycol) cross-linked by cyclodextrin.^7^ One of the most important natural polymers used in the fabrication of hydrogels is CS.^8–10^ It is a polysaccharide acquired by alkaline hydrolysis of chitin and is one of the most plenty natural polysaccharides exploited from the exoskeleton of crustaceans and the cell wall of fungi and insects.^11^ They have a large number of hydroxyl and amine functional groups in their structure that can bind to crosslinkers to form the hydrogel structure. The presence of hydrophilic amino and hydroxyl functional groups in the structure of CS causes water and biological fluids to be well absorbed in the hydrogel network.^12^ In addition, CS can be broken down by varied enzymes (mostly lysozyme) *in vivo* and easily be extruded or added to glycosaminoglycans and glycoproteins.^13^ All of these properties make CS a viable candidate for the development of new biomedical materials. Reportedly, polyvinyl alcohol is one of the oldest and most common synthetic polymers used to make hydrogels.^14^ In addition, SF as a natural protein is the main component of silk fibers that is extracted from silkworm cocoons. It is used frequently in biological application due to specific features such as high hemostatic, non-toxic, non-immunogenic, effective in the cell cycle. Also, it can also increase cell adhesion, fibroblast proliferation and cell growth, which is very effective and practical in tissue engineering, wound healing and drug delivery. Also, SF contains hydrophilic groups that can disperse well in water through hydrogen bonding with water molecules.^15^ This feature makes SF very efficient for hyperthermia cancer therapy, because the good dispersion of particles in aqueous environments is very effective on the efficiency of hyperthermia. One of the commonly used polymers in bioapplication is poly(vinyl alcohol) (PVA). PVA is a water-soluble linear polymer composed of partial or complete hydrolysis of acetate groups in polyvinyl acetate.^16^ The degree of hydrolysis determines the physical, chemical, and mechanical properties of PVA.^17^ As the degree of hydrolysis increases, the solubility in water decreases, and the more arduous it is to crystallize.^18^ PVA polymer has received attention in biomedical due to its special properties including biocompatibility, non-toxic, inflammatory, non-carcinogenic, and bioadhesive properties.^19^ Polymers composition with magnetic nanoparticles has many applications in biomedicine because it creates new materials with excellent properties that don't obtain by individual polymers.^20^ Magnetic iron oxides are common metal oxides with a crystal structure that has a cubic reverse spinel structure in which Fe^2+^ and Fe^3+^ occupy octahedral and quadrilateral lattices and are closed along with the [1,1,1] plane.^21^ Iron oxide nanoparticles have been used in most studies due to their biocompatibility, chemical stability, high magnetic sensitivity, high saturation magnet, and harmlessness.^22–31^ Among these, magnetite nanoparticles have been the most widely used iron oxide nanoparticles in biomedicine.^32,33^ The diverse application of magnetic nanoparticles makes them suitable for different fields of drug delivery, medicine, hyperthermia cancer therapy, electronics and contrast materials for magnetic resonance imaging (MRI). The small size of these nanoparticles gives them the ability to travel inside the cavity for drug delivery. In addition, they are specifically used as contrast materials for MRI. For *in vivo* applications, magnetic nanoparticles should be compatible with body fluids and non-toxic. Also, these nanoparticles tend to degrade inside the body. Therefore, placing them in a polymer matrix can be very useful.^34^ In this work, a biological system was constructed by low-toxicity materials such as chitosan hydrogel, SF, and PVA. Then, this nanobiocomposite was magnetized with Fe_3_O_4_ MNPs. The MNPs can be very effective in hyperthermia cancer therapy due to their high magnetic properties. These nanoparticles were encapsulated with natural polymers that are biocompatible to minimize the toxicity for *in vivo* application. All the used materials in the nanobiocomposite are hydrophilic and have good dispersibility in water due to the presence of hydrophilic functional groups in their structure, including hydroxyl groups (–OH), carbonyl groups (–C=O), carboxyl groups (–COOH), and amino groups (–NH_2_). Dispersibility in water is one of the effective factors in the efficiency of hyperthermia. Therefore, the use of hydrophilic materials in this nanobiocomposite is effective in increasing the efficiency of hyperthermia cancer therapy. Also, the presence of SF with unique capabilities in increasing adhesion and cell growth can prepare this nanobiocomposite for other biological applications, including tissue engineering and wound healing. Since mechanical properties are very important in biological applications, PVA polymer was used to improve the mechanical properties of the designed nanobiocomposite. In addition, Fe_3_O_4_ as magnetic nanoparticles was used to improve the magnetic property for hyperthermia application. The fabricated magnetic nanobiocomposite characterized with the variety analytical methods such as FT-IR, XRD, TGA, VSM, FE-SEM, and EDX. Also, the biological tests proved that this nanobiocomposite was hemocompatible and non-toxic for healthy cells. Also, it can be act as an effective compound for hyperthermia cancer therapy.

## 2. Experimental

### 2.1. General

All material that used in this work were prepared from Aldrich and Merck international companies. The deacetylation degree of CS was 75% and the molecular weight and degree hydrolysis of PVA are 44.05 g mol^−1^ and >98%, respectively. The prepared nanobiocomposite was investigated by versatile analysis such as FT-IR, XRD, FE-SEM, EDX, VSM, and TGA. The Fourier transform infrared spectroscopy (FT-IR) spectrum was performed by PerkinElmer Spectrum RX1 instrument. The X-ray diffraction analysis (XRD) analysis was done by Bruker device (D8 Advance model). FE-SEM images were taken by TESCAN (MIRA III model) device, Czech Republic. EDX analysis was applied by TESCAN MIRA II X-Max, France. As well as, VSM and TGA analysis was performed by LBKFB model-magnetic Kashan Kavir device and BahrSTA 504 under the argon atmosphere and the rate of 10 °C min^−1^, respectively. All analyses well proved the synthesis of bio-nanocomposite CS hydrogel–PVA/SF/Fe_3_O_4_. Based on the amount of saturated magnetism, the evaluation of hyperthermia application of nanocomposite synthesized was evaluated by system (NATSYCO, Iran) device.

### 2.2. Practical

#### 2.2.1. Preparation of terephthaloyl thiourea cross-linked CS hydrogel

Based on previous works,^26^ first, 1 M solution of ammonium thiocyanate and 0.5 M of terephthaloyl chloride in dichloromethane solvent were prepared. Then, the terephthaloyl chloride solution was added dropwise to the ammonium thiocyanate solution. After that, 1 ml of polyethylene glycol-400 was appended to the prepared mixture solution as a phase transfer catalyst and stirred for 2 h at room temperature. Next, the prepared white precipitate of ammonium chloride was separated from the gained yellow solution by filtration. Afterward, 3.22 g of CS powder was dissolved in 200 ml of acetic acid (1%) and added to the provided yellow solution, and stirred at 60 °C temperature for 2 h. In this step, the homogenous cross-linked CS hydrogel was prepared. Then, the saturated solution of sodium carbonate was used to neutralization fabricated hydrogel (pH = 7) and immersed in methanol for dehydration during the day.

#### 2.2.2. SF extraction

According to previous research,^35^ briefly, three silk cocoons were cut into small pieces. Then, they were added to 0.21 wt% boiled sodium carbonate solution and kept at 60 °C temperature for 2 h (at this stage, the glue-like sericin proteins were removed from cocoon pieces). Afterward, the degummed fibers were washed several times with distilled water and then dried at room temperature for 12 hours. Then, a 9.3 M LiBr solution was prepared and 10 times the weight of dried silk fibers was determined as the volume of LiBr solution. So, 6.19 ml of fresh LiBr solution (9.3 M, in H_2_O) was utilized to dissolve the weighted silk fibers (0.619 g) and stirred for 2 h. Henceforth, remaining LiBr in SF was removed by dialysis process (dialysis tubing cellulose membrane (14 000 Da)) for three days against water at room temperature. Eventually, extracted fibroin silk (SF) was kept at 4 °C for the other synthesis steps. To determine the weight of SF, 3 cm^3^ of SF solution was freeze-dried and 0.01 g of SF powder was obtained.

#### 2.2.3. Preparation of CS–SF hydrogel/PVA/Fe_3_O_4_ nanobiocomposite scaffold

In this step, 1 ml of SF was added to 1 ml of cross linked CS hydrogel and kept under stirring condition for 2 h at room temperature. Afterward, a solution of 0.05 (wt%) of polyvinyl alcohol was prepared and added to 1 ml of the mixture solution (CS–SF hydrogel) and stirred for 2 h at room temperature. Then, 0.97 g of FeCl_3_·6H_2_O, 0.44 g of FeCl_2_·4H_2_O powders and 40 ml of distilled water were added to 10 ml of the prepared mixture solution with its gel like foundation. Next, the mixture solution was stirred under N_2_ atmosphere and 10 ml 25% aqueous ammonia was appended drop by drop until pH ∼ 12 was obtained. After that, the mixture solution was stirred at 70 °C for 2 h. After the mixture was cooled, the black precipitate was collected by an external magnet and washed several times until it reached neutral pH (pH = 7).

#### 2.2.4. Hemolytic assay

In order to specify the potential lytic effects and the blood compatibility of nanobiocomposite on human erythrocytes, a red blood cells (RBCs) hemolytic assay was performed. First, RBCs were washed and diluted with physiological serum (0.9% w/v of NaCl, pH 7.0) in a ratio of 2 : 100. Then, 100 μl of it was poured onto a 96-well V-shaped bottom plate (Citotest, China) and each well received 100 μl of dispersed nanobiocomposite in NaCl 0.9% (0.25, 0.5, 0.75, 1 and 2 mg ml^−1^). Triton X-100 and physiological serum were applied as positive and negative controls, respectively. Thereafter, the plate was incubated for 2 h at 37 °C and then centrifuged at 2000 rpm for 10 minutes. Eventually, the supernatants were transferred to the flat bottom plate and the OD of each was determined at 405 nm using the ELISA reader (Biohit, Finland).^36,37^ The hemolysis ratio was calculated by following formula:^38^Hemolysis ratio (HR) = (TS − NC)/(PC − NC) × 100TS = test sample, NC = negative control, PC = positive control.

#### 2.2.5. MTT assay

MTT assay was used to measure the toxicity of our nanobiocomposite. For this purpose, the breast cancer cell line (BT549) and human embryonic kidney cell line (HEK293T) were furnished from the Pasteur Institute of Iran and cultured at 5 × 10^3^ cell per well in 96 well plate in the proper culture medium (DMEM/F12, 10% fetal bovine serum (FBS), and 1% pen–strep). Then, serially dilutions of nanobiocomposite (0.015, 0.031, 0.062, 0.125, 0.25, 0.5, 0.75, 1, 1.25, 1.5 and 1.75 mg ml^−1^) were added to each well and incubated for 48 h and 72 h. Cisplatin (Sigma-Aldrich, MO, United States) and the culture medium without any additive were also used as the positive and negative controls, respectively. The cells were then treated with 3-(4,5-dimethylthiazol-2-yl)-2,5-diphenyl tetrazolium bromide (MTT) solution (Sigma, USA) and incubated for 4 h at 37 °C. Next, 1% SDS was added to the wells and incubated for 16 h at 37 °C. Finally, the OD of samples was measured at 550 nm using a microplate reader spectrophotometer (BioTeK, USA). All tests were done in triplicate.^39,40^ The percentage of toxicity and cell viability were calculated as follows:^41^
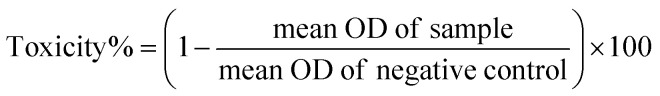
Viability% = 100 − toxicity%

#### 2.2.6. Statistical analysis

Statistical analysis for the comparison all biocompatibility and hemocompatibility results was accomplished by a *t*-test by SPSS Statistics 22.0 software (SPSS Inc. Chicago, IL, USA). The values of *P* ≥ 0.05 (*), *P* ≤ 0.05 (**) and *P* ≤ 0.001 (***) were considered as statistically insignificant, significant and very significant, respectively.

## 3. Result and discussion

**Scheme 1:**
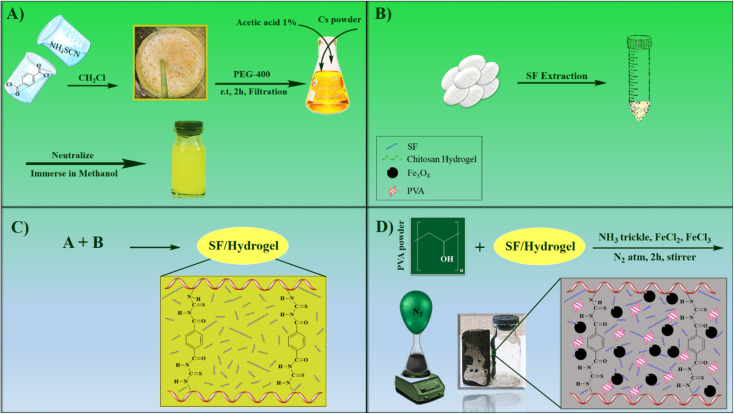
Synthetic process of the scaffold CS–SF hydrogel/PVA/Fe_3_O_4_ nanobiocomposite.

In this work, a magnetic nanocomposite consisting of biodegradable polymers with low toxicity was fabricated in four steps. First, the CS polymer filaments formed a hydrogel scaffold by the crosslinking agent terephthaloyl diisothiocyanate and then combined with the synthetic polymer polyvinyl alcohol and the natural protein SF. Finally, Fe_3_O_4_ magnetic nanoparticles were placed in the gel matrix. All synthesis steps are shown in Scheme 1. After the fabrication of the novel CS–SF hydrogel/PVA/Fe_3_O_4_ nanocomposite scaffold, its structural features and characteristics were identified by various analyzes. FT-IR analysis was taken to identify new functional groups of the nanobiocomposite structure. XRD analysis was performed to defining the crystalline phase of Fe_3_O_4_ in prepared nanobiocomposite. To determine the morphology and structure of novel CS–SF hydrogel/PVA/Fe_3_O_4_, the FE-SEM analysis was done. The other structural characteristics such as the elemental composition, thermal behavior and magnetic property was assessed by EDX, TGA and VSM analysis. All analyses well confirmed the synthesis of the new nanobiocomposite. In addition, hemolysis and MTT tests of the compound were performed to assess its toxicity to the body. Finally, hyperthermia application was appraised and all results were evaluated.

### 3.1. FT-IR spectroscopy and XRD analysis

**Fig. 1:**
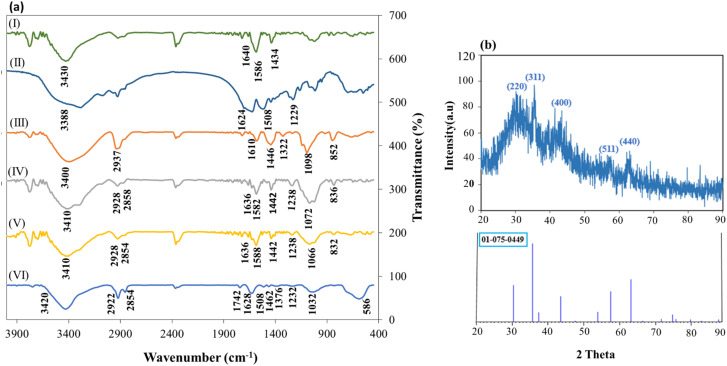
The FT-IR analysis (a) of CS (I), SF (II), PVA (III), CS–SF hydrogel (IV), CS–SF hydrogel/PVA (V), CS–SF hydrogel/PVA/Fe_3_O_4_ (VI) and XRD pattern of CS–SF hydrogel/PVA/Fe_3_O_4_ nanobiocomposite scaffold (b).

FT-IR spectroscopy was used to characterize CS (I), SF (II), PVA (III), cross-linked CS–SF hydrogel (IV), CS–SF hydrogel/PVA (V) and cross-linked CS–SF hydrogel/PVA/Fe_3_O_4_ nanobiocomposite (VI). In Fig. 1a(I), the presence of absorbance peak at 3430 cm^−1^ is assignable to stretching vibration of N–H and O–H bonds. Also, the appearance peaks at 1640, 1586 and 1434 cm^−1^ are attributed to the stretching vibrations of C=O bond (amide I), the bending vibrations of N–H bond (amide II) and the stretching vibrations of C–N bond (amide III), respectively.^42^ In Fig. 1a(II), three characteristic peaks, which have appeared at 1229, 1508, and 1624 cm^−1^ are related to the stretching vibration of C–N bond of amide III, N–H bending vibration of amide II, and the stretching vibration of carbonyl group in the SF structure. The spectrum of PVA has shown at the Fig. 1a(III). As can be seen, the absorbance peaks at 3400, 2937, 1610, 1446, 1322, 1098 and 852 cm^−1^ are assigned to the stretching vibrations of O–H bond, the asymmetric stretching vibration of CH_2_ bond, the stretching vibration of C=O bond, the bending vibrations of C–H bond, C–H deformation vibration, the stretching vibration of C–O acetyl groups and the stretching vibration of C–C, respectively.^43^Fig. 1a(IV) displayed the FT-IR spectrum of cross-linked CS–SF hydrogel. A broad bond was seen in the region of 3100 to 3600 cm^−1^ (3410 cm^−1^) was recognized for the stretching vibration of O–H and N–H bonds. The absorption peaks around of ∼2928 cm^−1^ and ∼2870 cm^−1^ was attributed to C–H symmetric and asymmetric stretching vibrations, respectively. The peak of C–H bending out of plane attributed to monosaccharide ring was appeared around ∼836 cm^−1^. Presence of two absorption bands at 1442 cm^−1^ and 1636 cm^−1^ was related to the C–N stretching vibration of amide III and amide I, respectively, which is similar to the CS spectrum. Also, the stretching vibration mode of C–O bond was developed as two peaks at ∼1072 cm^−1^ and ∼1026 cm^−1^. Then, the stretching vibration of C–O–C bridge bond was appeared at 1072 cm^−1^ that overlapped with the stretching vibration of C–O bond. An absorption band around 1632 cm^−1^ was assigned to the carbonyl bond (C=O), C=C bond and N–H bond of amine (II) of phenyl that overlapped. Absorption appeared peaks at ∼1442 and ∼1072 cm^−1^ were attributed to the –N–C–S– bending vibration and C=S stretching vibration bands. SF protein is usually identified by three distinct peaks in the FT-IR spectrum which was well seen in the Fig. 1a(IV). These absorption peaks appeared at ∼1636 cm^−1^, ∼1582 cm^−1^, and ∼1230 cm^−1^ that attributed to the sharp C=O stretching vibration of amide I, the N–H bending vibration of amide II, and the C–N stretching vibration of amide III, respectively.^35^ The peak of C–N stretching vibration, which has appeared at 1230 cm^−1^ is similar to the SF spectrum. The other peaks such as N–H bending vibration of amide II and C=O stretching vibration of amide I in the ligand structure have overlapped to the peaks attributed to C=O (amide I) and N–H (amide II) bonds in the SF structure. Fig. 1a(V) showed the FT-IR spectrum of cross-linked CS–SF hydrogel/PVA. All absorption peaks related to CS–SF hydrogel were well seen in this spectrum and the peaks of PVA were developed by the bands 832, 1036, 1744, 2924 and 3428 cm^−1^ that attributed to the vibration stretching of C–C in alkyl chain back bone, C–O stretching, C=O stretching, C–H of alkyl stretching state and OH vibrations, respectively.^43^ FT-IR spectrum of CS–SF hydrogel/PVA/Fe_3_O_4_ nanobiocomposite scaffold was demonstrated in Fig. 1a(VI). As can be seen, the fundamental absorption band and peaks of CS–SF hydrogel/PVA nanobiocomposite scaffold was indicated in FT-IR spectrum of fabricated magnetic nanocomposite. In addition to them, a strong absorption band around 586 cm^−1^ was became evident which was ascribed to the presence of Fe_3_O_4_ MNPs in the structure of cross-linked CS–SF hydrogel/PVA/Fe_3_O_4_ nanobiocomposite.^44^ X-ray diffraction (XRD) pattern of the prepared CS–SF hydrogel/PVA/Fe_3_O_4_ nanobiocomposite was taken to investigate the crystalline phase of composite components, as shown in Fig. 1b. As observed, a broad peak starts from region 2*θ* = 21° and continues to 2*θ* = 40°, which was related to the crystal structure of CS hydrogel and SF polymers. A considerable surface of hydrogen bonding in the CS powder was decreased after crosslinking through NH_2_ groups, so constituting a smaller part of the crystalline phase and a larger part of the amorphous phase. These results proved that the prepared polymer composite parts do not have a well-defined crystal structure compared to the inorganic part. The other peaks appeared at 2*θ* = 30.14, 35.44, 43.40, 57.31, and 63.13 and were relatively sharp. These peaks were related to the crystal structure of Fe_3_O_4_ nanoparticles and assigned with Miller indexes (220), (311), (400), (511), and (440), respectively (JCPDS no. 01-075-0449).^22,45–47^

### 3.2. EDX analysis

**Fig. 2:**
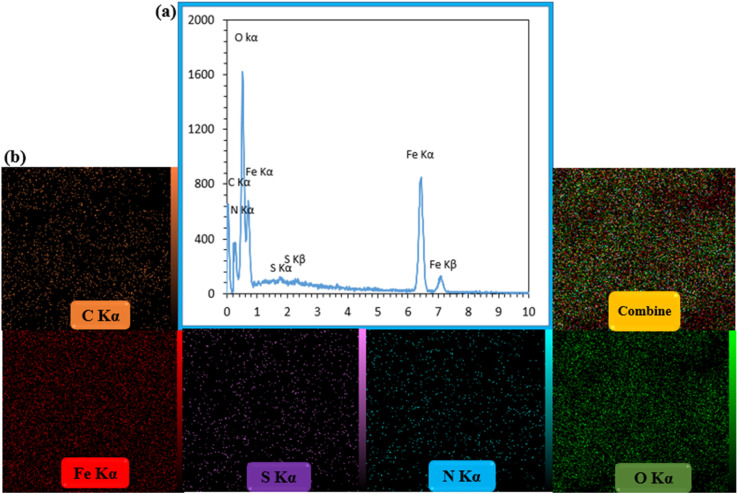
The EDX spectrum (a) and elemental mapping images of CS–SF hydrogel/PVA/Fe_3_O_4_ nanobiocomposite scaffold (b).

EDX analysis was taken of CS–SF hydrogel/PVA/Fe_3_O_4_ as a qualitative specification technique for the structural elements of various compounds that its results are shown in Fig. 2a. The constituent elements of the synthesized bionanocomposite were well observed. The peaks of nitrogen, oxygen and carbon related to the organic structures of CS hydrogel, fibroin silk and polyvinyl alcohol were identified. In addition, two peaks of sulfur in the terephthaloyl diisothiocyanate cross-linker were well observed. Also, two peaks of iron confirmed the presence of Fe_3_O_4_ NPs in the synthesized nanobiocomposite. Furthermore, the distribution of elements was well illustrated in the element mapping images (Fig. 2b).

### 3.3. FE-SEM imaging

**Fig. 3:**
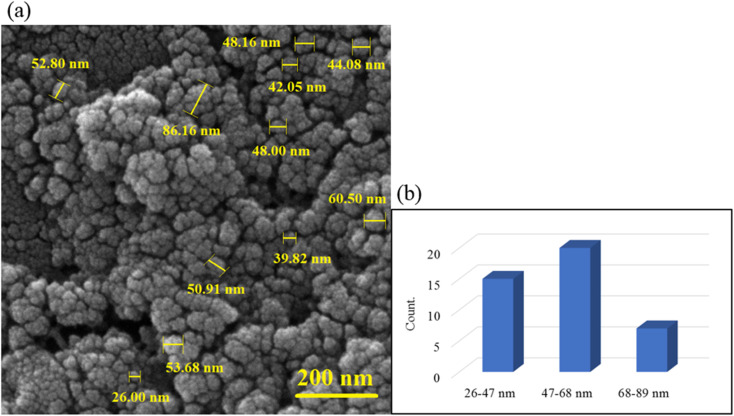
FE-SEM image of CS–SF hydrogel/PVA/Fe_3_O_4_ nanobiocomposite scaffold in magnification 200 nm (a) and the particle size histogram of CS–PVA hydrogel/SF/Fe_3_O_4_ magnetic nanobiocomposite (b).

The surface imaging results are shown in Fig. 3. As observed, the sphere structure was observed with an almost uniform particle size distribution. The uniform dispersion of magnetic nanoparticles in the gel matrix is effective in increasing the efficiency of hyperthermia. As can be seen, the Fe_3_O_4_ nanoparticles are well dispersed in the hydrogel matrix (Fig. 3a). The magnetization of hydrogel was performed *in situ*. When the Fe_3_O_4_ MNPs were being formed, they dispersed in the hydrogel matrix and formed a core–shell structure. The histogram of the particle size was examined in three ranges of 26–47, 47–68, and 68–89 nm. Most of the particles were between 47–68 nm and the particles were slightly dispersed.

### 3.4. Thermogravimetric and VSM analysis

**Fig. 4:**
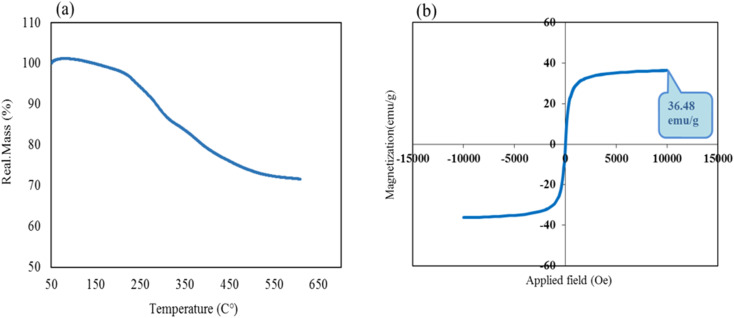
Thermogravimetric curve (a) and room-temperature magnetic-hysteresis curves of fabricated CS–SF hydrogel/PVA/Fe_3_O_4_ nanobiocomposite scaffold (b).

The thermal stability behavior of CS–SF hydrogel/PVA/Fe_3_O_4_ nanobiocomposite scaffold was investigated by TG analysis. As shown in Fig. 4a, first, a 1% weight gain from 50 to 145 °C was observed, which can be attributed to the effect of instrument buoyancy.^48^ After that, a reduction peak (∼4%) was started from almost 145 °C to 230 °C which was attributed to the evaporation of trapped water molecules. It is clear that the nanobiocomposite designed to approximately 230 °C has shown good thermal resistance and after that, it begins to lose a lot of weight. With increasing temperature to 600 °C, the fabricated composite lost almost 24% of its weight. Reportedly, at about 240 to 300 °C, the peptide bonds and the side chain groups of amino acid remnant of the SF structure are broken.^35^ Subsequently, increasing the temperature to 550 °C caused carbonization and oxidative decomposition of polymer chains of CS and PVA, respectively.^49^ The magnetic feature of the fabricated product was also scrutinized by vibrating-sample magnetometer (VSM) (Fig. 4b). According previous reports,^28^ magnetic saturation value of Fe_3_O_4_ MNPs was 76.20 amu g^−1^. By comparing the paramagnetic demeanor of Fe_3_O_4_ NPs with CS–SF hydrogel/PVA/Fe_3_O_4_, it is observed that the magnetic feature of Fe_3_O_4_ NPs was diminished about ∼40 emu g^−1^ after composition with CS–SF hydrogel/PVA/Fe_3_O_4_. Based on obtained results, the value of 36.48 emu g^−1^ as a saturation magnetization was estimated for this prepared nanobiocomposite which this amount was enough to get the desired results.

### 3.5. Hemocompatibility

**Fig. 5:**
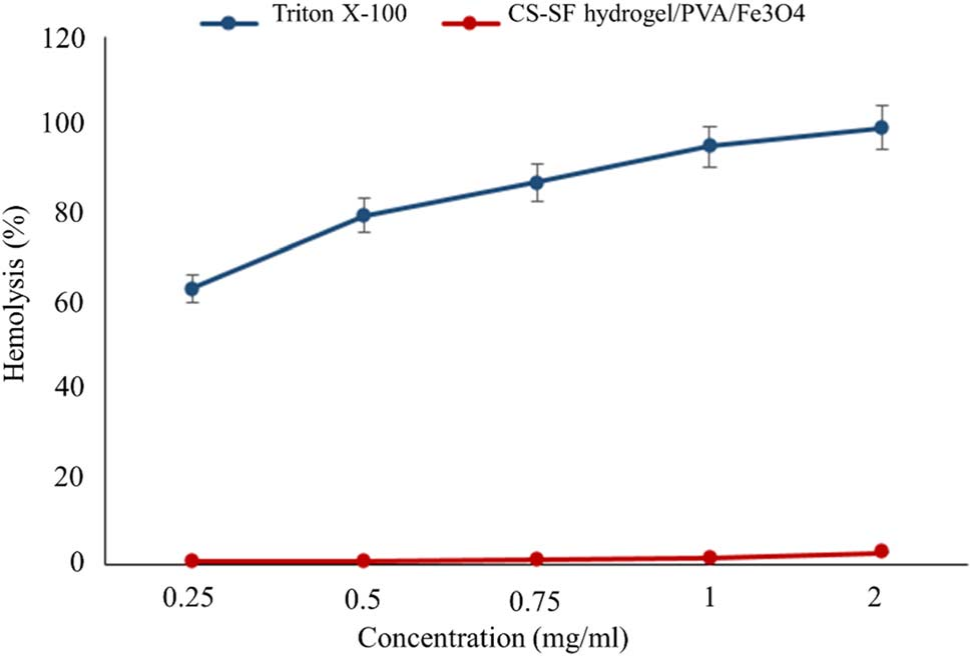
Hemolysis percentage graph of CS–SF hydrogel/PVA/Fe_3_O_4_ and Triton X-100 (positive control) at different concentrations (very significant compared to the positive control group, *P* ≤ 0.001).

According to the ISO standard (document 10993-5 1992), when a hemolysis index of a substance is less than 5%, it is considered safe.^50^ As can be seen in Fig. 5, at the highest concentration (2 mg ml^−1^), the hemolysis percentage is 2.73%, which proves that CS–SF hydrogel/PVA/Fe_3_O_4_ is an excellent hemocompatible nanobiocomposite and can have biomedical applications. Also, Triton X-100 (as a positive control) lysed almost all RBCs. The results are the mean of three independent experiments.

### 3.6. Toxicity test for normal and cancer cells

**Fig. 6:**
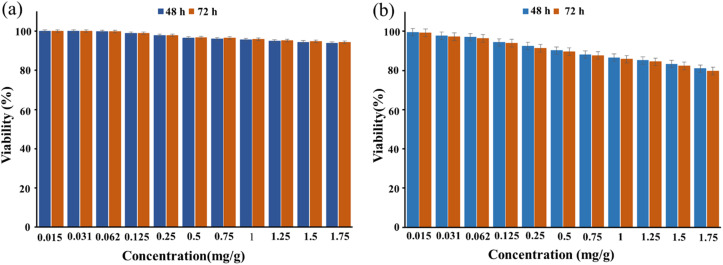
This illustration shows the viability percentage of HEK293T cells (a) BT549 cells (b) and after treatment with CS–SF hydrogel/PVA/Fe_3_O_4_ nanobiocomposite at days 2 and 3.

As shown in Fig. 6a, the survival rate of HEK293T cells after treatment with CS–SF hydrogel/PVA/Fe_3_O_4_ (1.75 mg ml^−1^) was 93.89% on the second day and 94.33% on the third day (insignificant compared to the negative control, *P* ≥ 0.05). So, this synthesized nanobiocomposite is biocompatible with this cell line.

Also, the viability percentage of BT549 cells treated with a concentration of 1.75 mg ml^−1^ of CS–SF hydrogel/PVA/Fe_3_O_4_ after 2 and 3 days was 80.94% and 79.73%, respectively (insignificant compared to the negative control, *P* ≥ 0.05) (Fig. 6b). Therefore, CS–SF hydrogel/PVA/Fe_3_O_4_ nanobiocomposite has been able to slightly inhibit the growth of BT549 cells and reduce their survival rate.

### 3.7. Application of prepared CS–SF hydrogel/PVA/Fe_3_O_4_ magnetic nanocomposite in hyperthermia process

Hyperthermia is a therapeutic procedure for cancer treatment which could be utilized as an appropriate replacement for conventional treatment techniques due to its lack of side effects. However, localizing heat to a desired site rather than heating the entire body appears to be more effective in this technique. Nanoparticles with magnetic materials are able to satisfy this requirement since they will operate as heat sources under the influence of an oscillating magnetic field. These magnetite nanoparticles (MNPs) increase the temperature of the surrounding fluid (41–46 °C) using hysteresis loss along with Néel and Brownian relaxations. Exciting MNPs by an oscillating magnetic field following delivering them to the tumor site would result in the destruction of cancer cells. The amount of released heat is directly affected by the properties of the magnetic field as well as the structure of the nanoparticles. On the one side, field strength and frequency and on the other side, size, shape, concentration and material of MNPs and the medium play a key role in therapeutic hyperthermia. The amount of heat generated is used to determine the efficiency of MNPs. A parameter called specific absorption ratio (SAR) is defined for this purpose which is the rate of the produced heat per unit mass.
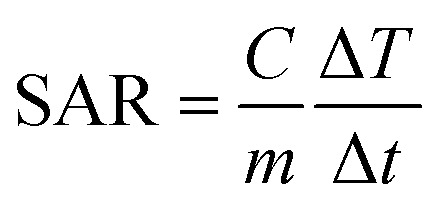
where *C* the specific heating capacity of the nanofluid and temperature change is denoted by Δ*T* created in the time interval of Δ*t*. *m* is the concentration of MNPs.

In order to evaluate the heating efficiency, an experimental test was conducted to obtain the heating profile of CS–SF hydrogel/PVA/Fe_3_O_4_ MNPs having a concentration of 1 mg ml^−1^. An oscillating magnetic field with constant intensity and different frequencies (100 kHz, 200 kHz, 300 kHz and 400 kHz) was imposed to the MNPs to investigate the impact of the field frequency as well and it was repeated 3 times for each frequency. Over a time, interval of 10 minutes from the beginning, the temperature was measured every 5 minutes. The initial temperature for each sample was 22.1 °C. According to Fig. 1a, as the process begins, the temperature increases. The maximum temperature rise is observed in the first 5 minutes which is 1.6 °C, 2.56 °C, 3.2 °C and 3.81 °C for the field frequency of 100 kHz, 200 kHz, 300 kHz and 400 kHz, respectively. The temperature rise in the first time interval increases as the field frequency grows and its maximum value was recorded at 400 kHz which is 3.81 °C. Over the entire time interval, the maximum obtainable temperature is 27.41 °C at 400 kHz which is 3.7 °C higher than the minimum one reported at 100 kHz. It is worth mentioning that in the second 5 minutes interval, the maximum temperature rise still occurs at 400 kHz which is about 1.5 °C.

**Fig. 7:**
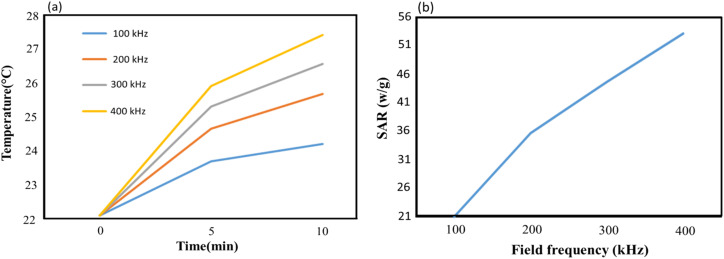
Heating profile of CS–SF hydrogel/PVA/Fe_3_O_4_ MNPs with concentration of 1 mg ml^−1^ in magnetic fields with various field frequencies (a) and maximum SAR as a function of field frequency for CS–SF hydrogel/PVA/Fe_3_O_4_ MNPs with concentration of 1 mg ml^−1^ (b).

The maximum SAR obtainable in the said time interval as a function of field frequency was depicted in Fig. 1b. Since SAR is proportional to the rate of temperature change, the steeper graph possesses greater SAR value. Therefore, the largest SAR value is 53.16 (W g^−1^) obtained at 400 kHz. It is anticipated that SAR increases as the field frequency grows. It experiences a rise from its lowest value, 21 (W g^−1^) at 100 kHz to its largest value, 53.16 (W g^−1^) at 400 kHz which is more than two times the lowest value. 35.72 (W g^−1^) and 44.65 (W g^−1^) was also calculated for SAR at the field frequencies of 200 kHz and 300 kHz, respectively. From 200 kHz to 400 kHz, SAR approximates to a linear function of frequency and in the entire range of the applied frequency, the overall average of SAR is calculated as 38.63 (W g^−1^) (Fig. 7).

## 4. Conclusions

In this study, a new nanobiocomposite based on low-toxicity materials was fabricated to improve biological properties. The natural polymer chains of CS were bonded together with terephthaloyl diisothiocyanate as a cross-linking agent to form a three-dimensional network hydrogel. Then, it was combined with the natural protein SF, a synthetic polymer of polyvinyl alcohol and Fe_3_O_4_ MNPs with low toxicity and high biocompatibility due to excellent biological properties. They created hybrid nanobiocomposites with great biological properties. Hemolysis and MTT tests were performed for the designed nanobiocomposite on human erythrocytes, the breast cancer cell line (BT549), and human embryonic kidney cell line (HEK293T). To explain more, the viability percentage of HEK293T healthy cells and BT549 cancer cells was 94.33% and 79.73% after three days in the presence of 1.75 mg ml^−1^ of the CS–SF hydrogel/PVA/Fe_3_O_4_ scaffold, respectively. Also, the hemolysis effect was 2.73% in the vicinity of the highest concentration prepared nanobiocomposite (2 mg ml^−1^). In addition, the synthesized nanobiocomposite for the treatment of hyperthermia cancer was studied and had remarkable results (SAR = 53.16 (W g^−1^)). All these brilliant results show that the designed nanobiocomposite is effective and efficient in cancer therapy. Presumably, the designed nanobiocomposite could be used in other biomedical applications, including drug delivery, tissue engineering, and wound healing due to its very low toxicity and excellent biological properties.

## Conflicts of interest

The authors declare no competing financial interest.

## Supplementary Material
