# Correction: Magnetized chitosan hydrogel and silk fibroin, reinforced with PVA: a novel nanobiocomposite for biomedical and hyperthermia applications

**DOI:** 10.1039/d5ra90112j

**Published:** 2025-10-08

**Authors:** Reza Eivazzadeh-Keihan, Zeinab Pajoum, Hooman Aghamirza Moghim Aliabadi, Adibeh Mohammadi, Amir Kashtiaray, Milad Salimi Bani, Banafshe Pishva, Ali Maleki, Majid M. Heravi, Mohammad Mahdavi, Elaheh Ziaei Ziabari

**Affiliations:** a Catalysts and Organic Synthesis Research Laboratory, Department of Chemistry, Iran University of Science and Technology Tehran 16846-13114 Iran maleki@iust.ac.ir +98-21-73021584 +98-21-73228313; b Department of Chemistry, School of Physics and Chemistry, Alzahra University PO Box 1993891176, Vanak Tehran Iran mmheravi@alzahra.ac.ir; c Advanced Chemical Studies Lab, Department of Chemistry, K. N. Toosi University of Technology Tehran Iran; d Department of Biomedical Engineering, Faculty of Engineering, University of Isfahan Isfahan Iran; e Endocrinology and Metabolism Research Center, Endocrinology and Metabolism Clinical Sciences Institute, Tehran University of Medical Sciences Tehran Iran momahdavi@sina.tums.ac.ir; f Department of Orthopedic Surgery, Rothman Institute, Thomas Jefferson University 125 South 9th Street, Suite 1000 Philadelphia PA 19107 USA

## Abstract

Correction for ‘Magnetized chitosan hydrogel and silk fibroin, reinforced with PVA: a novel nanobiocomposite for biomedical and hyperthermia applications’ by Reza Eivazzadeh-Keihan *et al.*, *RSC Adv.*, 2023, **13**, 8540–8550, https://doi.org/10.1039/D3RA00612C.

The authors regret that they omitted a citation to ref. 1 in their original article. This citation should be included in Section 3 (Result and discussion) before Section 3.1 (FT-IR spectroscopy and XRD analysis).

The authors wish to add the following paragraphs to the discussion section of the original article, to be read in Section 3 (Result and discussion) before Section 3.1 (FT-IR spectroscopy and XRD analysis), after the sentence “… Finally, hyperthermia application was appraised and all results were evaluated.” in order to clarify the novelty of this work over ref. 1.

In the previous work, we developed a multifunctional magnetic nanobiocomposite hydrogel based on graphene oxide (GO), which exhibited excellent properties for hyperthermia and biomedical applications due to its high thermal conductivity, mechanical reinforcement, and favorable interactions with the silk fibroin matrix.^1^

In contrast, the present study reported in *RSC Advances* focuses on polyvinyl alcohol (PVA) as a hydrophilic synthetic polymer with distinct physicochemical characteristics, in combination with a chitosan hydrogel/silk fibroin matrix. Various studies have demonstrated the preparation of functional magnetic nanoparticles coated with specific polymers, thermoresponsive copolymers, or polymeric composites for magnetic hyperthermia applications. Similarly, in these two research efforts, the polymeric component was modified—from graphene oxide to PVA—to explore biological properties, hyperthermia performance, and interactions within the chitosan hydrogel/silk fibroin composite matrix.

As clearly indicated, GO in the previous composite was replaced with PVA in the current study for several deliberate reasons. One key rationale was the replacement of GO with a biodegradable and low-toxicity polymer, as the conventional preparation of GO involves hazardous reagents and procedures. Specifically, the classical or modified Hummer’s method for graphite oxidation to GO requires concentrated sulfuric acid, strong oxidizing agents such as KMnO_4_, and often NaNO_3_ or alternative oxidizers. These reactions are highly exothermic, produce strong oxidizing conditions, and are quenched with H_2_O_2_, posing both immediate hazards (*e.g.*, explosion/fire risk, toxic gas emission) and generating toxic waste.^1^ In this context, the use of PVA is far safer and more environmentally friendly.

Furthermore, PVA offers unique biological and physicochemical properties in hydrogel systems. It is hydrophilic, biocompatible, non-toxic, non-carcinogenic, and exhibits low protein adhesion while forming excellent films, displaying high swelling capacity and notable mechanical strength.^2^ These features make PVA an attractive candidate for biomedical applications, including tissue engineering, wound healing, and drug delivery.^3^ For instance, PVA has been utilized in human brain tumor assessments, demonstrating its safety and clinical applicability.^4^ The hydroxyl groups in PVA allow extensive hydrogen bonding, enhancing hydrogel network stability and crosslinking potential.^5^ When combined with natural polymers such as chitosan and silk fibroin, PVA not only improves mechanical integrity and flexibility but also provides a supportive microenvironment that mimics extracellular matrix properties.^6^ Consequently, its integration into the chitosan hydrogel/silk fibroin system creates a composite with superior biological properties suitable for various biomedical applications, including drug delivery, magnetic hyperthermia-assisted therapy, wound healing, and tissue engineering, thereby paving the way for future biological studies.

Considering these advantages, we selected PVA as a reinforcing component in the designed system comprising chitosan, silk fibroin, and Fe_3_O_4_ magnetic nanoparticles, aiming to investigate its biological and hyperthermia properties within the composite framework. The incorporation of PVA was intended to enhance biocompatibility, swelling behavior, and mechanical stability of the hydrogel while maintaining biological safety. Thus, the rationale behind integrating PVA is rooted in its excellent biological and structural properties. Unlike GO, PVA does not impart thermal conductivity; instead, it primarily enhances mechanical elasticity and water absorption capacity, which are particularly advantageous in applications such as drug delivery and wound healing.

MTT assay results confirmed the high biocompatibility of the nanobiocomposite containing PVA, with HEK293T cell viability reaching 94.33% on day three following treatment with CS-SF/PVA/Fe_3_O_4_ hydrogel (1.75 mg mL^−1^). In contrast, GO exhibits significant dose-dependent toxicity even at low concentrations (10 μg mL^−1^), whereas PVA demonstrates high oral safety, minimal gastrointestinal absorption, and no accumulation *in vivo*, supporting its suitability as a biocompatible polymer for drug delivery systems.^7–9^

Additionally, PVA combined with magnetic nanoparticles offers multiple advantages for hyperthermia treatment. When applied as a coating on magnetic iron oxide nanoparticles, PVA: (1) protects the magnetic core from oxidation under harsh chemical or physiological conditions; (2) enhances colloidal stability and prevents particle aggregation, critical for effective *in vivo* performance, as PVA can maintain nanoparticle dispersion through hydrogen bonding with CS/SF, minimizing aggregation and improving saturation magnetization and heat generation efficiency.^4^ Indeed, the PVA-containing composite exhibited a saturation magnetization of ∼36.48 emu g^−1^, significantly higher than that of the GO-containing composite (∼17.09 emu g^−1^). (3) PVA enhances hydrophilicity and ensures a more uniform and stable dispersion in aqueous media, thereby improving the efficiency and reliability of hyperthermia applications.^10–12^ (4) PVA improves biocompatibility and reduces potential toxicity of magnetic nanoparticles, enhancing their efficacy and stability under physiological conditions, thereby minimizing systemic distribution risks and optimizing hyperthermia performance.

Taken together, for localized hyperthermia applications, the use of PVA as the third component in the nanocomposite is safer, simpler, and lower-risk compared to GO. While GO provides thermal conductivity benefits, it poses substantial environmental, biological, and synthetic challenges.

An independent expert has reviewed the associated raw data and explanation from the authors and deemed that a correction is appropriate in this case.

The Royal Society of Chemistry apologises for these errors and any consequent inconvenience to authors and readers.
